# The relationship between dexmedetomidine administration and prognosis in patients with sepsis-induced coagulopathy: a retrospective cohort study

**DOI:** 10.3389/fphar.2024.1414809

**Published:** 2024-07-23

**Authors:** Hongyu Huang, Qifei Li, Qingming Lin, Zheng Gong, Lujia Chen, Feng Chen, Xing Liao, Shirong Lin

**Affiliations:** ^1^ Shengli Clinical Medical College of Fujian Medical University, Fuzhou, Fujian, China; ^2^ Department of Emergency, Fujian Provincial Hospital, Fuzhou, Fujian, China; ^3^ Fujian Provincial Key Laboratory of Emergency Medicine, Fuzhou, Fujian, China; ^4^ Department of Medical Oncology, Clinical Oncology School of Fujian Medical University, Fujian Cancer Hospital, Fuzhou, Fujian, China

**Keywords:** dexmedetomidine, sepsis-induced coagulopathy, propensity score matching, (MIMIC)-IV database, 28-day hospital mortality

## Abstract

**Background:** This study aimed to investigate whether dexmedetomidine provides survival benefit in critically ill patients with sepsis-induced coagulopathy (SIC).

**Methods:** Patients with sepsis-induced coagulopathy admitted to the ICU were identified from the Medical Information Marketplace for Intensive Care (MIMIC)-IV database. They were divided into two groups: patients who started dexmedetomidine within 48 h of ICU admission and lasted for more than 4 h and patients who did not receive dexmedetomidine as a control group. The primary outcome was 28-day hospital mortality, the secondary outcome was in-hospital mortality, and the extended outcomes included duration of mechanical ventilation and vasopressor use, ICU stay, and hospital stay. Propensity score matching (PSM) analysis was used to match patients who received dexmedetomidine with those who did not, and multivariable Cox models and logistics models were used to account for baseline differences and unmeasured confounders. An external validation was performed with the Critical care database comprising patients with infection at Zigong Fourth People’s Hospital.

**Results:** After PSM, 592 patients who received dexmedetomidine were matched with 592 patients who did not receive dexmedetomidine. In the primary and secondary endpoints, dexmedetomidine was associated with a lower risk of 28-day hospital mortality (19.3% vs. 14.2%, hazard ratio (HR) 0.71; P = 0.020) and in-hospital mortality (22.3% vs. 16.4%, odds ratio (OR) 0.68; P = 0.017) in patients with SIC. Regarding the extended outcome, dexmedetomidine was also associated with a longer length of hospital stay (median 12.54 days vs. 14.87 days, P = 0.002) and longer ICU stay (median 5.10 days vs. 6.22 days, P = 0.009). In addition, the duration of mechanical ventilation was significantly increased in the dexmedetomidine group (median 41.62 h vs. 48.00 h, *p* = 0.022), while the duration of vasopressor use was not significantly different (median 36.67 h vs. 39.25 h, *p* = 0.194). Within 48 h of ICU stay, receiving a dose of dexmedetomidine greater than 0.474 μg/kg/h and continuous dexmedetomidine administration for 24–48 h may be associated with 28-day hospitalization outcomes in patients with SIC. External cohort validation also found that the use of dexmedetomidine after admission to the ICU can reduce 28-day mortality in patients with SIC.

**Conclusion:** Dexmedetomidine administration is associated with reduced 28-day hospital mortality and in-hospital mortality in critically ill patients with SIC, and these findings deserve further verification in randomized controlled trials.

## 1 Introduction

The Global Burden of Disease Study shows that sepsis affects at least 49 million patients each year and accounts for 19.7% of deaths worldwide (Rudd et al., 2020). Even with advances in medicine, more than 42% of sepsis patients in intensive care units (Fleischmann-Struzek et al., 2020) are still at high risk of death. 50%–70% of sepsis patients suffer from coagulopathy, which may lead to poor prognosis (Levi snd van der Poll, 2017). The mortality rate of sepsis-induced coagulopathy (SIC) is 23.1% (Iba et al., 2019), and the mortality rate of sepsis-related DIC is more than twice that of sepsis patients without DIC (Lyons et al., 2018). Given the key role of coagulation system dysfunction in sepsis, the DIC Scientific and Standardization Committee (SSC) introduced the term sepsis-induced coagulopathy (SIC) in 2017 (Iba et al., 2017) to accompany the third international consensus definition of sepsis (sepsis-3) (Singer et al., 2016). The SIC score includes the severity of thrombocytopenia, the international normalized ratio (INR) level, and the SOFA score, and is designed to detect early coagulation abnormalities for timely intervention.

Dexmedetomidine (DEX) is a potent agonist that selectively targets α2-adrenergic receptors. It is frequently used in the intensive care setting to induce sedation and provide pain relief for critically ill patients (Kawazoe et al., 2017). Different from traditional immunomodulatory treatments for sepsis such as corticosteroids, the number of emerging unconventional immunomodulatory treatments such as dexmedetomidine in sepsis is increasing in recent years (Slim, M. A et al., 2024). In the intensive care unit (ICU), the DESIRE randomized evolution (DESIRE) trial showed that dexmedetomidine was associated with lower mortality in patients with severe sepsis (Nakashima et al., 2020). In addition, another study found that dexmedetomidine was associated with improved renal function recovery and reduced 28-day mortality in patients with severe sepsis-related acute renal insufficiency (Hu et al., 2022). Animal studies have shown that dexmedetomidine can protect the vascular endothelial barrier function in septic rats, thereby reducing vascular leakage (Mei et al., 2021). In addition, dexmedetomidine has been shown to reduce the proinflammatory response triggered by lipopolysaccharide (Meng et al., 2020). Increasing evidence supports the anti-inflammatory properties of dexmedetomidine in various diseases (Ning et al., 2017; Kang et al., 2018).

The effect of dexmedetomidine on coagulation function is controversial, Studies have examined the impact of dexmedetomidine on coagulation function (Ma XF et al., 2023). Demonstrated that intervention with dexmedetomidine significantly improved coagulation dysfunction in patients undergoing radical gastrectomy under general anesthesia, leading to notable increases in prothrombin time, thromboxane B2, and fibrinogen levels. Similarly (Chen et al., 2018),investigated the role of dexmedetomidine in reducing coagulation activation following radical gastrectomy. They measured coagulation parameters pre- and post-surgery, and conducted thromboelastography on blood samples [including reaction time, clot formation time, and clot formation rate]. Post-surgery, the dexmedetomidine group exhibited significantly lower plasma concentrations of Thrombin-Antithrombin Complex and Fibrin Degradation Products compared to the control group. However, the clinical implications and broader impact of these findings warrant further detailed analysis. According to Shin et al. (2021), an increase in the concentration of dexmedetomidine leads to a hypercoagulable state in all coagulation pathways. Further research and detailed analysis are needed to fully understand how dexmedetomidine affects coagulation function.

There is growing evidence that dexmedetomidine provides critical protection against organ damage in various systems in sepsis by regulating inflammation and apoptosis (Kai et al., 2018; Sha et al., 2019). However, there is a lack of research on the effect of dexmedetomidine on the survival prognosis of patients with SIC. Dexmedetomidine has anti-inflammatory effects in various diseases (Ning et al., 2017; Kang et al., 2018) and may have potential effects on coagulation function. Therefore, this study aims to explore the potential correlation between the use of dexmedetomidine and the prognosis of patients with SIC through a large retrospective data set.

## 2 Materials and methods

### 2.1 Data

This study utilized data from the Multiparameter Intelligent Monitoring in Intensive Care (MIMIC-IV) database (Johnson et al., 2023), which includes ICU patient data from Beth Israel Deaconess Medical Center from 2008 to 2019. The creation and use of the MIMIC database was approved by the Institutional Review Board (IRB) of Beth Israel Deaconess Medical Center, affiliated with the Massachusetts Institute of Technology (MIT). Patient consent was not required as all protected personal information was de-identified and deleted. This retrospective observational study involved the extraction of clinical data from SIC patients using database management software and language tools. Data were then exported, processed, and analyzed using data analysis software to ensure that patient treatment was not compromised and remained safe. The author, Huang. HY, was certified and licensed by the Collaborative Institutional Training Initiative (CITI) to use the MIMIC-IV database in accordance with relevant regulations.

### 2.2 Population selection criteria

The study included patients diagnosed with sepsis-induced coagulopathy (SIC) within 24 h of intensive care unit (ICU) admission. Sepsis is defined as life-threatening organ dysfunction (Sepsis 3.0) (Singer et al., 2016) caused by an unbalanced response to infection, with confirmed or suspected infection and a sudden increase of two points or more in the total score of the Sequential Organ Failure Assessment (SOFA). The scoring system developed by Toshiaki Iba uses PT-INR, platelet count, and SOFA score level to identify SIC (Iba et al., 2017). Detailed criteria for the diagnosis of SIC can be found in Supplementary Table S1.

Patients with multiple admissions were included in the analysis based on their first admission only. Exclusion criteria included minors (<18 years old), pregnant women, ICU stay of less than 48 h, use of heparin after ICU admission, intravenous infusion of dexmedetomidine for less than 4 h, and use of dexmedetomidine for more than 48 h after ICU admission.

### 2.3 Data collection and definitions

This retrospective observational study was based on the Medical Information Mart for Intensive Care IV (MIMIC-IV) (version 2.2), Utilizing PostgreSQL software version 15.3 and Navicat Premium version 16, MIMIC-IV2.2 data information was extracted using structured query language. The code repository for this extraction can be found at https://github.com/MIT-LCP/mimic-iv/tree/master/concepts. The demographic information of the patients included age, gender, and ethnicity, as well as body weight, urinary output, and various findings from lab tests (such as WBC count, platelet count, hemoglobin, hematocrit, anion gap, bicarbonate, BUN, creatinine, glucose, sodium, potassium, INR, PT), physiological measurements (pulse rate, MAP, respiratory rate, oxygen saturation), existing health conditions (like diabetes, high blood pressure, heart disease, lung disease, kidney disease, liver disease, cancer history), usage of blood pressure medication, propofol, fentanil, midazolam, CRRT, ventilator support, SIC severity score, length of stay in hospital and ICU, and survival data. Additionally, clinical severity scales like the SOFA score and the Simplified Acute Physiology Score II (SAPS II) were also collected.

Duration of vasopressor use refers to the duration of norepinephrine, epinephrine, and vasopressor use during the patient’s ICU stay. Detailed information about dexmedetomidine included the drug name, dose, route of administration, and start and end time. To assess the potential dose-dependent effect of dexmedetomidine on the prognosis of patients with SIC, dexmedetomidine dose was expressed as μg/kg/hour.

### 2.4 Outcomes

The primary outcome was 28-day hospital mortality, the secondary outcome was in-hospital mortality, and the extended outcomes included duration of mechanical ventilation and vasopressor use, ICU stay, and hospital stay.

### 2.5 Statistical analysis

In this study, the missing proportion of each variable was less than 5%, and detailed data can be found in Supplementary Table S2. Multiple imputation was performed using the mice package in R to ensure data completeness and availability (Allison, 2000). SIC patients who received dexmedetomidine formed the experimental group, while those who did not receive dexmedetomidine belonged to the control group. Mann-Whitney *U* test was used to analyze non-normally distributed continuous variables, and the results were expressed as median and interquartile range. Chi-square test was used to compare categorical variables between the two groups.

In our study, we used propensity score matching (caliper value of 0.05) to reduce the differences in baseline characteristics between the two groups. Subsequently, we calculated the standardized mean difference (SMD) to evaluate the effectiveness of PSM in mitigating these differences (Harder et al., 2015). To show the frequency of 28-day death in SIC patients, we used Kaplan-Meier curves. Cox regression models were used to investigate the association between dexmedetomidine administration and the outcome of 28-day in-hospital mortality, and the log-rank test was used to assess the difference. Cox proportional hazards models were used to calculate the hazard ratio (HR) and 95% confidence interval (CI) between dexmedetomidine and endpoints, and some models were adjusted. In the univariate analysis, confounding variables selected based on *p*-value < 0.05, including clinically relevant variables and prognostic-related variables, were also included in the multivariate model: Model 1: unadjusted; Model 2: adjusted for age, gender, ethnicity; Model 3: adjusted for age, gender, ethnicity, vasoactive drugs, CRRT, and SIC score, propofol, midazolam, fentanyl, tumors, liver diseases, heart, resprate, SpO2, aniongap, bicarbonate, BUN, creatinine. In addition, subgroup analysis of SIC patients was performed based on gender, age (≤60 years and >60 years), SOFA score (≤8 points and ≥8 points), SIC score, diabetes, hypertension, mechanical ventilation, and vasopressor use. The hazard ratio (HR) and 95% confidence interval (CI) were calculated for each subgroup.

Statistical analysis was performed using R version 4.3.1, and statistical significance was set at P < 0.05.

### 2.6 External validation

External validation was performed using the Critical care database comprising patients with infection at Zigong Fourth People’s Hospital version 1.1 (Zhang et al., 2018). The database at Zigong Fourth People’s Hospital in Sichuan Province, China, contains records of 2,790 infected ICU patients from January 2019 to December 2020. Approval for establishing this database was granted by the Ethics Committee of Zigong Fourth People’s Hospital (approval number: 2020-065) (Zhang et al., 2018). As this study was retrospective in nature, individual patient consent was not necessary. All authors obtained permission to access the database.

The external validation set included 2,790 infected patients diagnosed with sepsis in the intensive care unit (ICU). To ensure an adequate number of SIC patients in the validation set, we relaxed the exclusion criteria, including minors (<18 years old), ICU stays of less than 48 h, use of heparin post-ICU admission, and non-SIC population. To mitigate the impact of a small sample size of in-hospital deaths on the external validation model, we opted for 28-day death as the survival outcome instead of limiting it to in-hospital deaths. To minimize confounding factors, we utilized Kaplan-Meier curves and Cox regression models to investigate the relationship between dexmedetomidine administration and 28-day death outcomes.

## 3 Results

### 3.1 Patient characteristics

**FIGURE 1:**
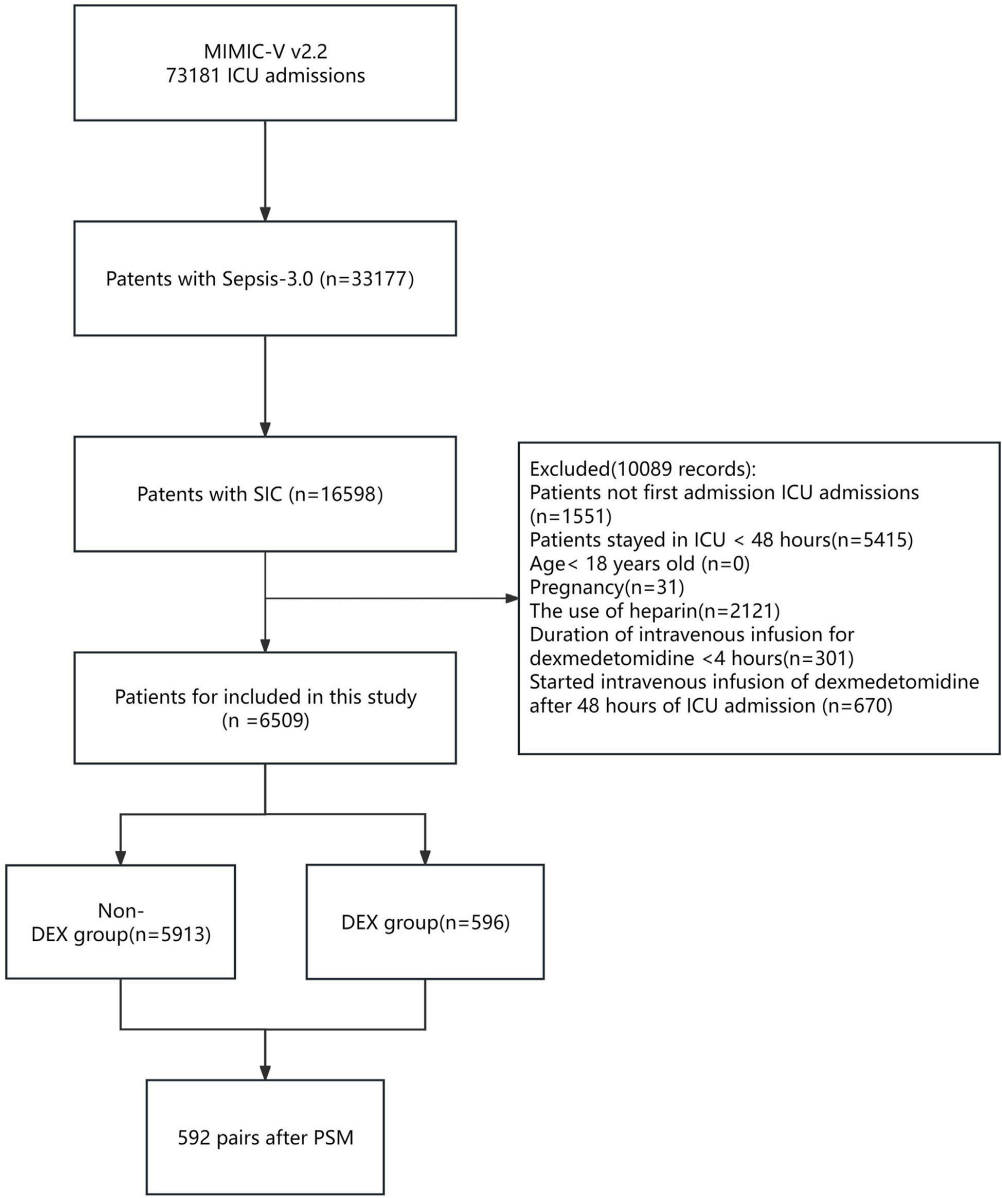
Flowchart of the study. Abbreviations: MIMIC-IV; Medical Information-Mart for Intensive Care IV; ICU: intensive care unit; SIC: sepsis-induced coagulopathy. DEX: dexmedetomidine. PSM: propensity score matching.

During the study period, a total of 16,598 critically ill patients with SIC were included (Figure 1). Based on the exclusion criteria, 6,509 patients were eligible for analysis. Among them, there were 596 cases in the dexmedetomidine group (DEX group) and 5,913 cases in the non-dexmedetomidine group (Non-DEX group).

There were significant differences between the DEX and non-DEX groups in terms of age, sex, race and weight, and urine output (Table 1). The DEX group had higher SOFA scores, more patients with SIC scores of 5-6, and higher usage rates of mechanical ventilation and vasopressors as well as propofol, remifentanil, and midazolam on the first day of ICU admission. Was higher, while there was no significant difference in the use of continuous renal replacement therapy (CRRT) between the two groups. A higher proportion of people in the non-DEX group had conditions such as kidney disease and tumors compared with the DEX group. After propensity score matching, 592 patients who received dexmedetomidine were matched with 592 patients who did not receive dexmedetomidine. Matched individuals exhibited uniform distribution of baseline characteristics, with Standardized Mean Difference (SMD) below 10% for all variables (Supplementary Table S1; Supplementary Figures S1-S3).

**TABLE 1:** Baseline characteristics of the two groups before and after propensity score matching.

Characteristics	Non-DEX group (n = 5,913)	DEX group (n = 596)	SMD	*p*	Non-DEX group (n = 592)	DEX group (n = 592)	SMD	*p*
Age (years)	69.76 [59.04, 79.81]	65.34 [55.21, 74.84]	0.28	<0.001	65.92 [56.15, 76.44]	65.36 [55.47, 74.84]	0.044	0.295
Gender [female, n (%)]	2,371 (40.1)	195 (32.7)	0.154	0.001	188 (31.8)	195 (32.9)	0.025	0.709
Urine output (mL)	1365.00 [780.00, 2,150.00]	1470.00 [899.00, 2,188.50]	0.075	0.013	1420.00 [853.50, 2,121.25]	1447.50 [858.75, 2,185.00]	0.01	0.666
Weight (kg)	79.75 [67.60, 94.90]	83.82 [71.60, 99.25]	0.172	<0.001	83.00 [68.80, 99.25]	83.80 [71.25, 99.10]	0.012	0.323
SOFA score	8.00 [5.00, 11.00]	9.00 [7.00, 12.00]	0.388	<0.001	10.00 [6.00, 12.00]	9.00 [7.00, 12.00]	<0.001	0.84
SAPSII score	42.00 [34.00, 52.00]	42.00 [34.00, 52.25]	0.051	0.473	43.00 [34.00, 54.00]	42.00 [34.00, 52.25]	0.027	0.504
Ethnicity, n (%)			0.158	0.001			0.087	0.327
White	4102 (69.4)	394 (66.1)			407 (68.8)	392 (66.2)		
Black	656 (11.1)	50 (8.4)			37 (6.2)	50 (8.4)		
Other	1155 (19.5)	152 (25.5)			148 (25.0)	150 (25.3)		
Interventions in the first 24 h, n (%)								
Mechanical ventilation	3049 (51.6)	513 (86.1)	0.803	<0.001	512 (86.5)	509 (86.0)	0.015	0.866
Vasopressor use	609 (10.3)	86 (14.4)	0.126	0.002	93 (15.7)	85 (14.4)	0.038	0.569
CRRT	509 (8.6)	47 (7.9)	0.026	0.6	42 (7.1)	47 (7.9)	0.032	0.659
Propofol	3033 (51.3)	500 (83.9)	0.743	<0.001	501 (84.6)	496 (83.8)	0.023	0.75
Midazolam	1734 (29.3)	200 (33.6)	0.091	0.035	219 (37.0)	198 (33.4)	0.074	0.224
Fentanyl	1971 (33.3)	280 (47.0)	0.281	<0.001	283 (47.8)	277 (46.8)	0.02	0.771
Comorbid, n (%)								
Hypertension	2,155 (36.4)	233 (39.1)	0.055	0.217	234 (39.5)	232 (39.2)	0.007	0.953
CHF	2,399 (40.6)	222 (37.2)	0.068	0.125	215 (36.3)	221 (37.3)	0.021	0.763
COPD	1621 (27.4)	162 (27.2)	0.005	0.942	161 (27.2)	158 (26.7)	0.011	0.896
Rheumatic disease	237 (4.0)	18 (3.0)	0.054	0.283	14 (2.4)	18 (3.0)	0.042	0.591
Liver disease	1518 (25.7)	146 (24.5)	0.027	0.563	140 (23.6)	146 (24.7)	0.024	0.734
Diabetes	1917 (32.4)	181 (30.4)	0.044	0.329	194 (32.8)	181 (30.6)	0.047	0.453
Renal disease	1763 (29.8)	154 (25.8)	0.089	0.047	156 (26.4)	154 (26.0)	0.008	0.947
Tumor	847 (14.3)	62 (10.4)	0.119	0.01	75 (12.7)	62 (10.5)	0.069	0.276
Vital signs in the first 24 h								
Heart rate (bpm)	86.80 [76.47, 99.71]	85.40 [77.62, 97.11]	0.051	0.342	85.92 [75.79, 97.75]	85.47 [77.65, 97.11]	0.009	0.581
MAP (mmHg)	73.48 [68.23, 79.49]	74.71 [69.88, 81.16]	0.15	<0.001	74.28 [69.97, 80.69]	74.68 [69.88, 81.05]	0.013	0.844
Resp rate (bpm)	19.07 [16.74, 22.24]	18.96 [16.66, 21.09]	0.102	0.051	18.54 [16.33, 21.57]	18.96 [16.66, 21.09]	0.038	0.335
SpO2 (%)	97.52 [96.00, 98.75]	98.00 [96.57, 99.00]	0.284	<0.001	98.11 [96.80, 99.16]	98.00 [96.58, 99.00]	0.036	0.232
Laboratory tests in the first 24 h								
Hematocrit (%)	26.50 [23.10, 31.00]	25.90 [22.87, 30.70]	0.052	0.14	25.90 [22.60, 30.13]	25.85 [22.80, 30.63]	0.03	0.814
Hemoglobin (×10^12/L)	8.80 [7.60, 10.20]	8.60 [7.50, 10.20]	0.068	0.052	8.60 [7.60, 10.00]	8.60 [7.50, 10.20]	0.02	0.916
Platelets (×10^9/L)	119.00 [77.00, 180.00]	104.00 [73.00, 146.00]	0.207	<0.001	108.00 [70.75, 157.25]	104.00 [73.00, 146.00]	0.051	0.532
WBC (×10^9/L)	9.10 [6.10, 12.90]	9.10 [6.40, 12.40]	0.027	0.892	9.10 [5.88, 12.60]	9.10 [6.40, 12.40]	0.035	0.531
Anion gap (mEq/L)	13.00 [11.00, 15.00]	12.00 [10.00, 15.00]	0.153	<0.001	12.00 [10.00, 15.00]	12.00 [10.00, 15.00]	0.011	0.66
Bicarbonate (mEq/L)	21.00 [18.00, 24.00]	21.00 [18.00, 23.00]	0.055	0.201	21.00 [18.00, 24.00]	21.00 [18.00, 23.00]	0.09	0.074
BUN (mg/dL)	23.00 [15.00, 39.00]	18.50 [13.00, 30.00]	0.231	<0.001	20.00 [14.00, 32.00]	18.50 [13.00, 30.00]	0.018	0.077
Creatinine (mg/dL)	1.10 [0.80, 1.80]	1.00 [0.70, 1.50]	0.178	0.002	1.00 [0.70, 1.50]	1.00 [0.70, 1.50]	0.028	0.688
Sodium (mEq/L)	137.00 [134.00, 139.00]	138.00 [135.00, 140.00]	0.193	<0.001	138.00 [135.00, 140.00]	138.00 [135.00, 140.00]	0.044	0.665
Potassium (mEq/L)	3.90 [3.50, 4.30]	3.90 [3.50, 4.30]	0.095	0.058	3.95 [3.58, 4.30]	3.90 [3.50, 4.30]	0.027	0.947
INR (ratio)	1.40 [1.20, 1.80]	1.30 [1.20, 1.60]	0.233	<0.001	1.30 [1.20, 1.70]	1.30 [1.20, 1.60]	0.015	0.474
PT (sec)	15.80 [13.70, 20.00]	14.40 [13.00, 17.30]	0.231	<0.001	14.85 [13.10, 18.12]	14.40 [13.00, 17.30]	0.008	0.139
Glucose (mg/dL)	131.67 [113.00, 160.62]	131.93 [118.49, 161.00]	0.014	0.077	133.69 [117.46, 166.49]	131.97 [118.49, 161.05]	0.019	0.693
SIC score, n (%)			0.108	0.045			0.082	0.368
4	2,895 (49.0)	260 (43.6)			275 (46.5)	258 (43.6)		
5	1537 (26.0)	169 (28.4)			146 (24.7)	167 (28.2)		
6	1481 (25.0)	167 (28.0)			171 (28.9)	167 (28.2)		

Abbreviations: PSM: propensity score matching; DEX: dexmedetomidine; SMD: standardized mean difference, SOFA: sequential organ failure assessment, SAPS II: Simplified Acute Physiology Score II,ICU: Intensive Care Unit,MAP: Mean Arterial Pressure,WBC: White Blood Cell Count,INR: International Normalized Ratio,PT: prothrombin time, APTT: Activated Partial Thromboplastin Time,SIC: Sepsis-Induced Coagulopathy, CRRT, Continuous Renal Replacement Therapy; Resp Rate, Respiratory Rate; SpO2, saturation of peripheral oxygen; BUN, Blood Urea Nitrogen.

### 3.2 Association between dexmedetomidine and primary and secondary outcomes

COX regression and logistic regression models were used to investigate the association between dexmedetomidine administration and 28 in-hospital mortality and in-hospital mortality before propensity score matching (PSM). The analysis showed that DEX group was associated with a reduced risk of 28-day in-hospital death compared with non-DEX group: unadjusted model: [18.7% vs. 14.3%, HR, 0.72 (0.58–0.90, *p* = 0.004)], partially adjusted model: [HR, 0.73 (0.58–0.91, *p* = 0.005)] and fully adjusted model: [HR, 0.78 (0.62–0.97, *p* = 0.028)] (Table 2). The risk of in-hospital death was reduced with dexmedetomidine: unadjusted model: [21.1% vs. 16.4%, OR, 0.74 (0.59–0.92, *p* = 0.008)], partially adjusted model: [OR, 0.72 (0.57–0.90, *p* = 0.005)] and fully adjusted model: [OR, 0.76 (0.60–0.98, *p* = 0.031)] (Table 2).

**TABLE 2:** Survival results of dexmedetomidine and non-user groups in SIC patients before PSM, after PSM.

Categories	28-day hospital mortality	In-hospital mortality
Before PSM	HR (95% CI,*p*-value)	OR (95% CI,*p*-value)
Model1	0.72 (0.58–0.90, *p* = 0.004)	0.74 (0.59–0.92, *p* = 0.008)
Model2	0.73 (0.58–0.91, *p* = 0.005)	0.72 (0.57–0.90, *p* = 0.005)
Model3	0.78 (0.62–0.97, *p* = 0.028)	0.76 (0.60–0.98, *p* = 0.031)

After PSM	HR (95% CI,*p*-value)	OR (95% CI,*p*-value)
Model1	0.70 (0.53–0.93, *p* = 0.013)	0.68 (0.51–0.91, *p* = 0.010)
Model2	0.68 (0.52–0.91, *p* = 0.008)	0.68 (0.50–0.91, *p* = 0.009)
Model3	0.71 (0.53–0.95, *p* = 0.020)	0.68 (0.49–0.93, *p* = 0.017)

Model 1: unadjusted.

Model 2: adjusted for age, gender, ethnicity.

Model 3: adjusting for confounding variables selected based on *p*-value <0.05 in univariate analysis, including age, gender, ethnicity, vasoactive drugs, CRRT, and SIC, score, propofol, midazolam, fentanyl, tumors, liver diseases, heart, resprate,SpO2,aniongap, bicarbonate,BUN, creatinine.

Abbreviations: SIC: Sepsis-Induced Coagulopathy; HR, hazard ratio; OR, odds ratio; CI, confidence interval. PSM: propensity score matching; DEX: dexmedetomidine; ICU: Intensive Care Unit,SpO2: oxygen saturation; CRRT, Continuous Renal Replacement Therapy; Resp Rate, Respiratory Rate; BUN, Blood Urea Nitrogen.

**FIGURE 2:**
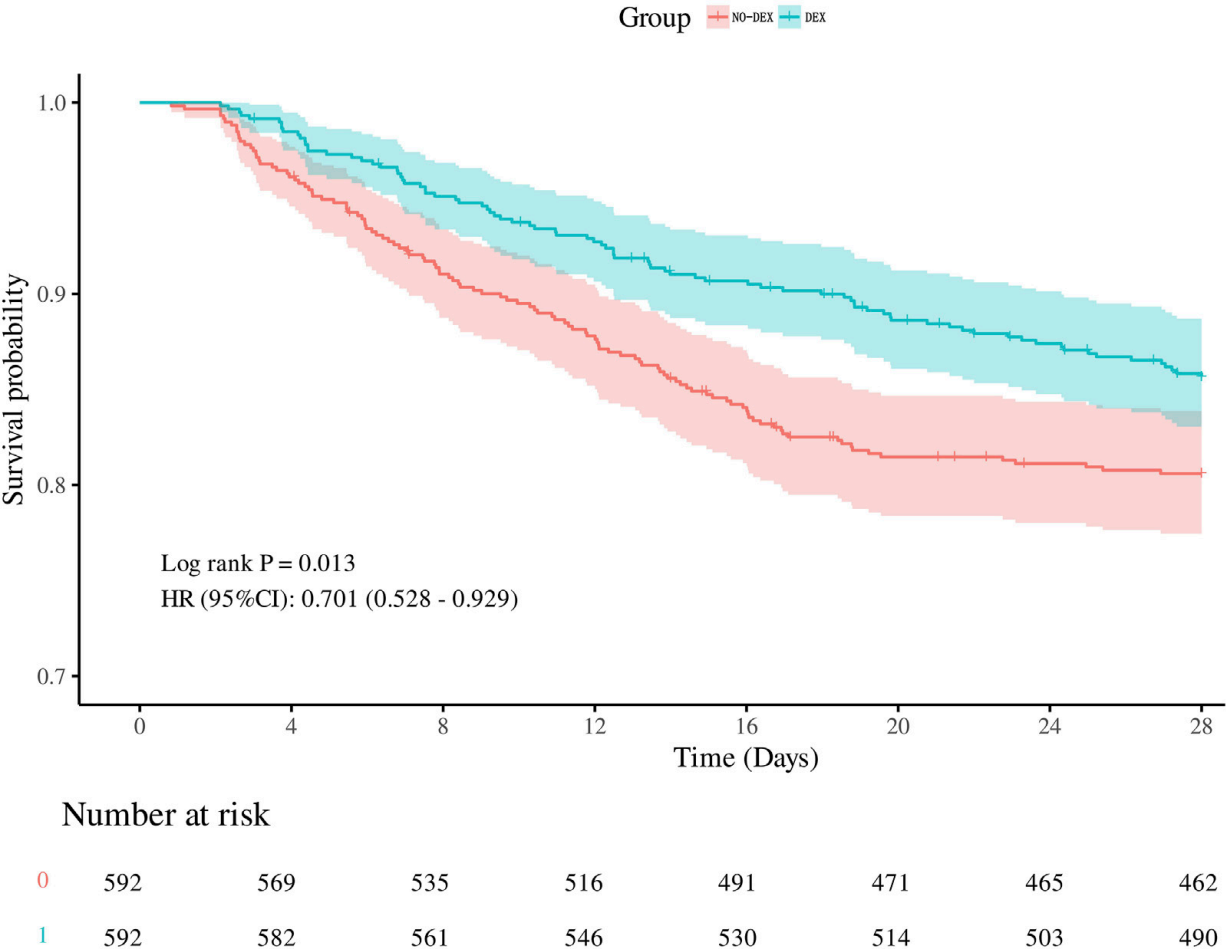
Kalpan-Meier survival curves between the two groups showing the 28-day risk of death in patients with SIC. Dexmedetomidine users are represented by the blue line, and non-dexmedetomidine users are represented by the red line.

After performing propensity score matching (PSM), Figure 2 shows the Kaplan-Meier curve, indicating that DEX group had a significantly higher 28-day survival rate than non-DEX group (HR, 0.701 (0.528–0.929), log-rank test: *p* = 0.013). Consistent with previous PSM results, the dexmedetomidine group was associated with a reduced risk of 28-day death: unadjusted model: [HR, 0.70 (0.53–0.93, *p* = 0.013)], partially adjusted model: [HR, 0.68 (0.52–0.52–0.91, *p* = 0.008)] and the fully adjusted model: [HR, 0.71 (0.53–0.95, *p* = 0.020)]. The risk of in-hospital death was reduced with dexmedetomidine: unadjusted model: [OR, 0.68 (0.51–0.91, *p* = 0.010)], partially adjusted model: [OR, 0.68 (0.50–0.91, *p* = 0.009)] and complete model Adjusted model: [OR,0.68 (0.49–0.93, *p* = 0.017)].

To assess the robustness of our findings to potential unmeasured or residual confounders, we performed a sensitivity analysis using E values to assess sensitivity to unmeasured confounders (https://www.evalue-calculator.com/evalue/). In the present study, our findings are robust, with a hazard ratio (HR) of 0.71 for dexmedetomidine treatment *versus* 28-day in-hospital mortality in patients with SIC, unless there are unmeasured or residual confounding covariates for 28 The relative risk of day-to-day in-hospital death must be greater than 1.85 to affect the observed hazard ratio for 28-day in-hospital mortality.

### 3.3 Association between dexmedetomidine and expanded outcomes

Before propensity score matching (PSM), dexmedetomidine use was associated with longer hospital stay (median 11.02 days vs. 14.89 days, *p* < 0.001) and longer ICU stay (median 4.23 days vs. 6.26 days, *p* < 0.001) correlation. In addition, the duration of mechanical ventilation was significantly longer in the dexmedetomidine group (median 38.00 h vs. 48.00 h, *p* < 0.001), and the duration of vasopressor use was significantly longer (median 33.70 h vs. 39.42 h, *p* < 0.001). *p* < 0.001) (Table 3).

**TABLE 3:** The association between dexmedetomidine administration and clinical outcomes in SIC patients.

Categories	Before PSM			After PSM		
Comprehensive results	Non-DEX group (n = 5,913)	DEX group(n = 596)	p	Non-DEX group (n = 592)	DEX group(n = 592)	p
28-day hospital mortality, n(%)	1107 (18.7)	85 (14.3)	0.009	114 (19.3)	84 (14.2)	0.024
In-hospital mortality, n(%)	1248 (21.1)	98 (16.4)	0.009	132 (22.3)	97 (16.4)	0.012
Mechanical ventilation duration (h)	38.00 [15.66, 90.58]	48.00 [21.00, 122.00]	<0.001	41.62 [15.52, 110.23]	48.00 [21.00, 121.33]	0.022
Vasopressor use duration (h)	33.70 [12.23, 71.63]	39.42 [18.00, 92.45]	<0.001	36.67 [13.73, 81.95]	39.25 [17.86, 92.15]	0.194
The length of hospital stay (d)	11.02 [6.85, 18.78]	14.89 [9.02, 22.70]	<0.001	12.54 [7.63, 20.88]	14.87 [8.98, 22.70]	0.002
The length of ICU stay (d)	4.23 [2.91, 7.46]	6.26 [3.88, 11.09]	<0.001	5.10 [3.27, 10.21]	6.22 [3.87, 11.09]	0.009

Abbreviations: SIC: Sepsis-Induced Coagulopathy; PSM: propensity score matching; DEX: dexmedetomidine; ICU: intensive care unit.

After propensity score matching (PSM), dexmedetomidine use was associated with longer hospital stay (median 12.54 days vs. 14.87 days, *p* = 0.002) and longer ICU stay (median 5.10 days vs. 6.22 days, *p* = 0.009) related. In addition, the duration of mechanical ventilation was significantly longer in the dexmedetomidine group (median 41.62 h vs. 48.00 h, *p* = 0.022), while there was no significant difference in the duration of vasopressor use (median 36.67 h vs. 39.25 h, *p* = 0.194) (Table 3).

### 3.4 Subgroup analysis

**FIGURE 3:**
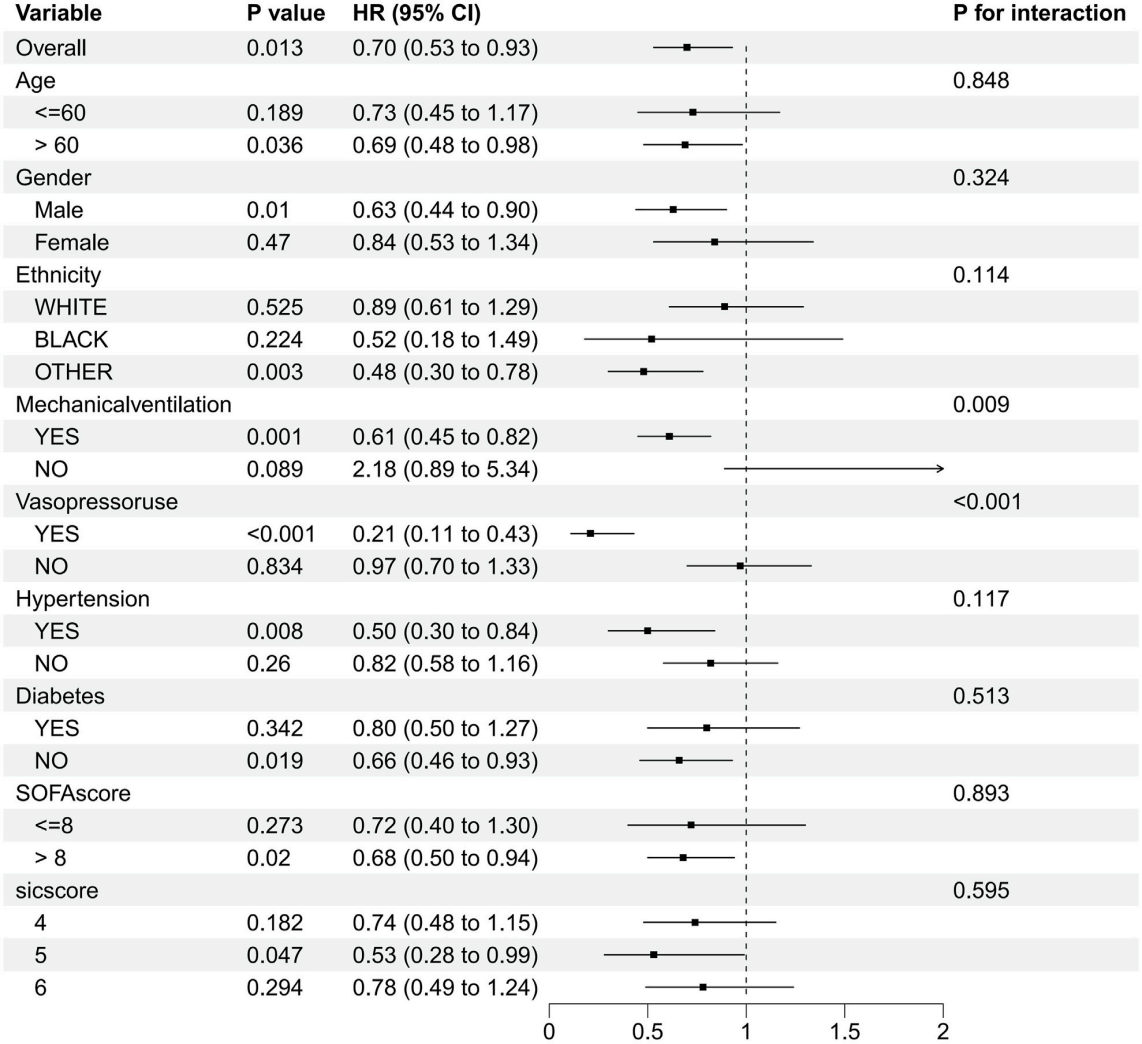
Subgroup analysis of the relationship between dexmedetomidine and 28-day in-hospital mortality as shown in forest plot.

SIC patients were categorized into various subgroups based on age, sex, race, comorbidities (hypertension, diabetes), mechanical ventilation, vasoactive medications, SOFA score, and SIC score. The forest plot (Figure 3) depicts the effect of dexmedetomidine on 28-day in-hospital mortality in patients with SIC. Our subgroup analysis showed that dexmedetomidine had a significant effect on 28-day in-hospital mortality in different patient subgroups. It is worth noting that patients over 60 years old (HR 0.69, 95% CI 0.48–0.98, *p* = 0.036), male patients (HR 0.63, 95% CI 0.44–0.90, *p* = 0.01), and patients of other races (HR 0.48, 95% CI 0.30–0.78, *p* = 0.003), as well as patients on ventilators (HR 0.61, 95% CI 0.45–0.82, *p* = 0.001), and patients on vasoactive drugs (HR 0.21, 95% CI 0.11–0.43, *p* < 0.001), patients with hypertension (HR 0.50, 95% CI 0.30–0.84, *p* = 0.008), non-diabetic patients (HR 0.66, 95% CI 0.46–0.93, *p* = 0.019), patients with SOFA score greater than 8 (HR 0.68, 95% CI 0.50–0.94, *p* = 0.02), and patients with SIC score 5 (HR 0.53, 95% CI 0.28–0.99, *p* = 0.047) showed a significant protective effect. In contrast, patients under 60 years old (HR 0.73, 95% CI 0.45–1.17, *p* = 0.189), female patients (HR 0.84, 95% CI 0.53–1.34, *p* = 0.47), and white patients (HR 0.89, 95% CI 0.61–1.29, *p* = 0.525), black patients (HR 0.52, 95% CI 0.18–1.49, *p* = 0.224), patients not receiving mechanical ventilation (HR 2.18, 95% CI 0.89–5.34, *p* = 0.089), not using Patients with vasoactive drugs (HR 0.97, 95% CI 0.70–1.33, *p* = 0.834), patients with diabetes (HR 0.80, 95% CI 0.50–1.27, *p* = 0.342), and patients with SOFA score less than eight points (HR 0.72, 95% CI 0.40–1.30, *p* = 0.273) and patients with SIC scores of four and 6 (HR 0.74, 95% CI 0.48–1.15, *p* = 0.182 and HR 0.78, 95% CI 0.49–1.24, *p* = respectively 0.294) did not show significant protective effects. In summary, the subgroup analysis of this study showed the potential protective effect of dexmedetomidine in different subgroups. The different effects between different subgroups suggest the heterogeneity of our study population, and dexmedetomidine may be valuable in personalized treatment strategies for patients with septic coagulopathy.

### 3.5 Dose-response and duration-response relationships between dexmedetomidine administration and survival outcomes

The median duration of dexmedetomidine administration ranged from 25.58 h. When comparing different duration ranges, it was found that short-term (4–24 h) use of dexmedetomidine had no significant impact on survival outcomes. Dexmedetomidine administered continuously over 24–48 h was associated with a reduced risk of 28-day in-hospital death and in-hospital mortality. For prolonged use (more than 48 h) of dexmedetomidine, although in-hospital mortality improved, the effect on 28-day mortality was not significant (Table 4).

**TABLE 4:** Duration-response and Dose-response relationships between dexmedetomidine administration and outcomes.

Categories		28-day hospital mortality	In-hospital mortality
Dexmedetomidine duration (h)	N (%)	HR (95% CI,*p*-value)	OR (95% CI,*p*-value)
Without dexmedetomidine	592 (50.0%)	1	1
4–24	282 (23.8%)	0.71 (0.48–1.06, *p* = 0.096)	0.90 (0.58–1.39, *p* = 0.637)
24–48	133 (11.2%)	0.49 (0.28–0.86, *p* = 0.013)	0.54 (0.33–0.89, *p* = 0.015)
>48	177 (14.9%)	0.85 (0.58–1.24, *p* = 0.405)	0.61 (0.38–0.98, *p* = 0.040)

Dexmedetomidine dose (μg/kg/h)	N (%)	HR (95% CI,*p*-value)	OR (95% CI,*p*-value)
Without dexmedetomidine	592 (50.0%)	1	1
<0.474	197 (16.6%)	0.87 (0.60–1.28, *p* = 0.495)	0.90 (0.58–1.39, *p* = 0.637)
0.474–0.710	198 (16.7%)	0.60 (0.38–0.96, *p* = 0.032)	0.54 (0.33–0.89, *p* = 0.015)
>0.710	197 (16.6%)	0.65 (0.42–1.00, *p* = 0.048)	0.61 (0.38–0.98, *p* = 0.040)

Abbreviations: HR, hazard ratio; OR, odds ratio; CI, confidence interval. Cox regression and Logistics regression was used to estimate the impact of dexmedetomidine use on mortality outcomes, adjusting for confounding variables selected based on *p*-value <0.05 in univariate analysis, including age, gender, ethnicity, vasoactive drugs, CRRT, and SIC, score, propofol, midazolam, fentanyl, tumors, liver diseases, heart, resprate,SpO2,aniongap, bicarbonate,BUN, creatinine.

The median dose rate of dexmedetomidine was 0.572 μg/kg/h, with a tertile dose range (33%–66%) of 0.474–0.710 μg/kg/h. Dexmedetomidine greater than 0.474 μg/kg/h was observed to be associated with a reduced risk of 28-day in-hospital death and reduced in-hospital mortality compared with the non-DEX group (Table 4).

### 3.6 External validation

A total of 234 patients were included in the external validation cohort. Patient characteristics are shown in the (Supplementary Table S1). We also found that the use of dexmedetomidine may prolong the length of hospital stay and ICU stay. The Kaplan-Meier curve showed that the 28-day survival rate in the DEX group was significantly higher than that in the non-DEX group (HR, 0.492 (0.278–0.871), log-rank test: *p* = 0.013) (Supplementary Figure S5). After adjusting for age, gender, propofol, and sufentanil, dexmedetomidine was also associated with a reduced 28-day mortality in sic patients (HR 0.49, 95% CI 0.27–0.89, *p* = 0.019) (Supplementary Table S2).

## 4 Discussion

In this retrospective cohort study, Our results showed that the use of dexmedetomidine was associated with decreased 28-day hospital mortality and in-hospital mortality in critically ill patients with sepsis-induced coagulopathy (SIC). In terms of the extended outcomess, the use of dexmedetomidine may lead to prolonged ICU and hospital stays and mechanical ventilation, but may not increase the duration of vasopressor use. In addition, considering the dose and duration of dexmedetomidine, our results showed that dexmedetomidine administered for 24–48 h and dexmedetomidine doses greater than 0.474 μg/kg/h were associated with reduced 28-day hospital mortality risk and hospital mortality in patients with SIC.In the external cohort validation set, it was also found that the use of dexmedetomidine after ICU admission could reduce the 28-day mortality of SIC patients.

Before this study, there were no studies on the correlation between dexmedetomidine and coagulation abnormalities in patients with sepsis. Our study is the first to suggest that dexmedetomidine may improve the prognosis of patients with sepsis-induced coagulopathy. The precise biological mechanism underlying the relationship between dexmedetomidine and sepsis-induced coagulation remains uncertain. One potential explanation is that dexmedetomidine plays a role in immune modulation, influencing the progression of sepsis-related coagulopathy *via* various biological pathways, such as anti-inflammatory responses and endothelial protection. Previous research has demonstrated that aside from its sedative properties, dexmedetomidine exhibits anti-inflammatory effects in both preclinical and clinical settings (Gao et al., 2019; Yu et al., 2021). The interaction between inflammatory response and coagulation in sepsis is complex (Li et al., 2020), and inflammation may trigger coagulation activation. Dexmedetomidine may indirectly improve coagulation function by inhibiting the release of inflammatory markers and reducing platelet activation (Kawazoe et al., 2017). Endothelial injury is a crucial aspect of sepsis-induced coagulopathy (Dolmatova et al., 2021; Mao et al., 2021). In animal sepsis models, dexmedetomidine has shown a notable protective effect on endothelial barrier function by upregulating intercellular junction proteins, enhancing transendothelial electrical resistance, and reducing vascular endothelial cell permeability (She et al., 2021). Protecting the endothelium can help prevent organ microthrombosis and sepsis-related coagulopathy (La Mura et al., 2022). However, the lack of data on inflammatory responses has hindered the verification of this theory, and further confirmation is needed in future studies.

Our results showed that dexmedetomidine reduced the 28-day hospital mortality and in-hospital mortality of critically ill patients with SIC. The definition of PT-INR level was based on the SIC scoring system developed by Toshiaki Iba. Previous studies have shown that the use of heparin can reduce the 28-day and in-hospital mortality of sepsis coagulopathy (Peng et al., 2021). Therefore, our study excluded patients who received heparin after ICU admission to minimize the impact of confounding factors on the outcomes. This retrospective study limited the use of dexmedetomidine to within 48 h after ICU admission to reduce bias, and sedative analgesics such as propofol, midazolam, and remifentanil were considered as confounders, and their effects on survival prognosis and length of hospital stay were mitigated by propensity scores. A multi-model multi-factor regression analysis was conducted, revealing that dexmedetomidine significantly reduced 28-day hospital mortality and in-hospital mortality both before and after PSM. Additionally, dexmedetomidine may prolong hospital stay, ICU stay, and mechanical ventilation time, We found similar results in external validation. As observed in the study by Hu et al. (Hu et al., 2022). The prolonged hospital stay may be due to the mortality-reducing effect of dexmedetomidine, which prolongs the treatment and recovery period. Some studies (Shan et al., 2022) have shown that dexmedetomidine can activate a highly selective α2-adrenergic receptor pathway, thereby reducing adrenal sympathetic overactivity. Experimental data (Cioccari et al., 2020) show that dexmedetomidine (DEX) can increase vasopressor responsiveness and reduce the need for catecholamines in septic shock. However, our study did not find a significant difference in the duration of vasopressor use. Therefore, further prospective research is necessary to investigate whether dexmedetomidine can decrease the requirement for vasopressors in SIC patients.

A recent study (Zhao et al., 2024) showed that the dose and timing of dexmedetomidine administration were associated with reduced 28-day mortality in septic patients requiring mechanical ventilation. Therefore, we further explored the correlation of dexmedetomidine dose and duration with prognosis in patients with SIC. Animal clinical trials conducted by Li, S et al. in 2015 demonstrated (Li, S et al, 2015) that DEX decreases the secretion of cytokines (TNF-α, IL-6) following endotoxin injection, and dexmedetomidine reduces endotoxemia in a dose-dependent manner in a rat model of induced shock. Our study consistently found that increasing the dose of dexmedetomidine above 0.474 μg/kg/h can lower the risk of 28-day in-hospital mortality and in-hospital mortality in patients with SIC. Moreover, we observed that continuous administration of dexmedetomidine for 24–48 h was linked to a decreased risk of 28-day in-hospital mortality and in-hospital mortality. However, when dexmedetomidine was used for over 48 h, although in-hospital mortality improved, the effect on 28-day mortality was not significant. It is important to note that escalating doses of dexmedetomidine may lead to adverse reactions like hypotension and bradycardia (Song et al., 2014), as well as potential effects such as prolonged hospitalization. Therefore, it is recommended that continuous use for 24–48 h or dexmedetomidine at a dose ranging from 0.474 to 0.710 μg/kg/h may offer the most benefits for patients with SIC. Subsequent research should focus on investigating the optimal dose and duration of dexmedetomidine in patients with SIC.

Sepsis is a severe, rapidly progressive and highly heterogeneous disease, and there is a need to focus on the classification of sepsis patients to guide precise therapeutic intervention. For example, (Zhang et al., 2018), explored multiple clusters Methods Establishing clinical subphenotypes of patients at risk for postoperative sepsis can help target therapy to improve the efficacy of this specific group. Another study by Zhang et al. (2018) also identified four types of sepsis. Subtypes, the four subtypes showed different mortality outcomes and responses to fluid resuscitation, with the coagulopathic subtype showing the highest mortality associated with comorbidity with other organ dysfunction. As a subtype of sepsis, SIC is also affected by the heterogeneity of the study population. In order to further explore the heterogeneity, we conducted subgroup analysis. Subgroup analysis results showed that dexmedetomidine was more significant in reducing the risk of 28-day in-hospital death in some subgroups, including elderly patients aged 60 and above, men, non-black and white patients of other races, patients with hypertension, and those without Patients with diabetes, those with a SOFA score of more than 8, those on ventilators, those requiring vasoactive drugs, and those with a SIC score of five had a reduced risk of death. The reason may be that age is a major risk factor for multi-organ failure in patients with sepsis (Inata et al., 2018), and dexmedetomidine may regulate the enhanced sympathetic nervous system function in sepsis (Sun et al., 2019; Tao et al., 2022) to improve prognosis, thereby inhibiting organ damage in elderly patients with SIC and reducing mortality. SCI patients of different genders may have different immune responses. Dexmedetomidine has shown potential efficacy in improving the immune response of male SIC patients, thereby improving the prognosis of male SIC patients. Different racial groups have different genetic backgrounds, which may affect their metabolism and response to drugs. Our study found that SIC patients in other racial groups (including Asians or other non-white and non-black races) are more likely to benefit from dexmedetomidine. On-treatment benefit, our external validation set from the Chinese cohort supports this argument, dexmedetomidine is associated with reduced 28-day mortality in patients with Sic Asian descent (HR 0.49, 95% CI 0.27–0.89, *p* = 0.019). Patients with hypertensive SIC may benefit from the modulation of sympathetic nervous system function during sepsis by dexmedetomidine, thereby stabilizing the cardiovascular status of hypertensive patients and improving prognosis. Non-diabetic patients may exhibit more stable drug metabolism than diabetic patients, contributing to the stable reduction of inflammatory mediators by dexmedetomidine and reducing the risk of death from septic coagulopathy. SIC patients with a SOFA score greater than eight indicate severe organ dysfunction, and dexmedetomidine may help improve the survival rate of patients with multiple organ dysfunction. Interaction analysis showed that dexmedetomidine had a significant effect on the protective effects of mechanical ventilation and vasoactive drugs. Dexmedetomidine significantly reduced the risk of death in patients receiving mechanical ventilation (HR (95% CI) 0.61 (0.45–0.82), *p* = 0.001), but not in patients not receiving mechanical ventilation. There was a significant effect in patients using vasoactive drugs (HR (95% CI) 0.21 (0.11–0.43), *p* < 0.001), whereas there was no significant change in patients not using vasoactive drugs (HR (95% CI) 0.97 (0.70–1.33), *p* = 0.834). This difference may be attributed to the presence of severe respiratory and circulatory failure in mechanically ventilated individuals and in SIC patients taking vasoactive drugs. Dexmedetomidine is believed to reduce the inflammatory response in sepsis, protect multiple organ functions, and thereby improve the prognosis of SIC patients with severe respiratory failure and circulatory failure. The DESIRE trial (Ohta et al., 2020) showed that dexmedetomidine improved outcomes in patients with sepsis requiring mechanical ventilation. Furthermore, studies by Zhao et al. (2024), Morelli et al. (2019) showed that compared with other sedative drugs, dexmedetomidine reduced 28-day mortality in mechanically ventilated patients with severe sepsis rate and reduced catecholamine requirements in patients with septic shock. Dexmedetomidine may improve prognosis by inhibiting inflammatory markers and reducing oxidative stress (Kawazoe et al., 2017), thereby improving vasopressin responsiveness (Cioccari et al., 2020) and alleviating patients’ coagulation disorders. Dexmedetomidine treatment may have important implications for 28-day in-hospital mortality in patients with SIC in patients receiving mechanical ventilation and vasoactive drugs. The SIC scoring system, including platelet count, prothrombin time (PT)-INR, and sequential organ failure assessment (SOFA) score, has higher sensitivity in predicting 28-day mortality in patients with SIC, interestingly our study It was found that the prognosis of patients with a SIC score of five was significantly improved compared with other SIC scores. Patients with a SIC score of five indicated that they had developed severe coagulation dysfunction. At this time, there is greater room for improvement with the participation of dexmedetomidine in immunomodulatory intervention, at this stage, dexmedetomidine, through its anti-inflammatory and sedative effects, may effectively reduce coagulation abnormalities and inflammation and avoid further organ damage. There may also be different SIC score subphenotypes that affect the observed results, but further exploration needs to be combined with multifactor analysis, and further prospective studies are needed to explore the role of dexmedetomidine in reducing mortality in patients with different SIC score subphenotypes. Potential.

Some limitations must be acknowledged in this study. First, this study is limited by use of the MIMIC-IV database, which only contains information on critically ill patients admitted from 2009 to 2018. This may be inconsistent with the most recent sepsis definition developed in 2016. However, we endeavored to identify patients according to the latest diagnostic criteria for SIC and sepsis (Sepsis-3). Second, as a retrospective analysis using the MIMIC-IV repository, there were significant differences between the groups in year of admission and initial patient characteristics. In the prematched sample, individuals in the DEX group were older, had higher SOFA scores, and were more likely to receive vasoactive medications and mechanical ventilation. Despite careful propensity score matching and multivariable analyses, residual confounding may still exist. Therefore, caution should be used in interpreting these results due to limited variables and significant heterogeneity. Third, performing a large number of subgroup analyzes may increase the likelihood of false positive results, even if the sample size of each group is large. The study was conducted in a single center and focused on a European and American population, which highlights the need for external validation from other populations. Future studies should consider including cohorts from Asian populations in order to conduct multi-center trials and validate the results. Fourth, while our study focused on external validation within the Asian population, we aimed to ensure a sufficient sample size by implementing relatively lenient exclusion criteria and using 28-day mortality as the primary outcome measure. Nonetheless, the final sample size remained small, potentially impacting the validity of the external validation study. Lastly, due to the retrospective nature of our study design, further confirmation of our research findings is warranted through future prospective studies.

## 5 Conclusion

The administration of dexmedetomidine may improve 28-day in-hospital survival and hospital survival in sepsis-induced coagulopathy. These findings could potentially inform clinical decision-making regarding the use of dexmedetomidine, but further validation is required through future randomized controlled trials.

## Data Availability

The datasets presented in this study can be found in online repositories. The names of the repository/repositories and accession number(s) can be found below: Publicly available datasets were analyzed in this study. This data can be found here: https://physionet.org/content/mimiciv/2.2/ (certification number: 56149575).

## References

[B1] Allison P. D. (2000). Multiple imputation for missing data: A cautionary tale. Sociological Methods and Research 28 (3), 301–309. 10.1177/0049124100028003003

[B2] Chen Z. Shao D. H. Mao Z. M. Shi L. L. Ma X. D. Zhang D. P. (2018). Effect of dexmedetomidine on blood coagulation in patients undergoing radical gastrectomy under general anesthesia: A prospective, randomized controlled clinical trial. Medicine 97 (27), e11444. 10.1097/MD.0000000000011444 29979445 PMC6076139

[B3] Cioccari L. Luethi N. Bailey M. Shehabi Y. Howe B. Messmer A. S. Proimos H. K. Peck L. Young H. Eastwood G. M. Merz T. M. Takala J. Jakob S. M. Bellomo R. (2020). The effect of dexmedetomidine on vasopressor requirements in patients with septic shock: a subgroup analysis of the Sedation Practice in Intensive Care Evaluation [SPICE III] Trial. Critical Care 24 (1), 441. 10.1186/s13054-020-03115-x 32678054 PMC7367420

[B4] Dolmatova E. V. Wang K. Mandavilli R. Griendling K. K. (2021). The effects of sepsis on endothelium and clinical implications. Cardiovascular Research 117 (1), 60–73. 10.1093/cvr/cvaa070 32215570 PMC7810126

[B5] Fleischmann-Struzek C. Mellhammar L. Rose N. Cassini A. Rudd K. E. Schlattmann P. (2020). Incidence and mortality of hospital- and ICU-treated sepsis: results from an updated and expanded systematic review and meta-analysis. Intensive Care Medicine 46, 1552–1562. 10.1007/s00134-020-06151-x 32572531 PMC7381468

[B6] Gao J. Sun Z. Xiao Z. Du Q. Niu X. Wang G. Chang Y. W. Sun Y. Sun W. Lin A. Bresnahan J. C. Maze M. Beattie M. S. Pan J. Z. (2019). Dexmedetomidine modulates neuroinflammation and improves outcome via alpha2-adrenergic receptor signaling after rat spinal cord injury. British Journal of Anaesthesia 123 (6), 827–838. 10.1016/j.bja.2019.08.026 31623841 PMC6883489

[B7] Harder V. S. Stuart E. A. Anthony J. C. (2015). Propensity score techniques and the assessment of measured covariate balance to test causal associations in psychological research. Psychological Methods 20 (3), 234–249. 10.1037/a0019623 PMC2936698 20822250

[B8] Hu H. An S. Sha T. Wu F. Jin Y. Li L. (2022). Association between dexmedetomidine administration and outcomes in critically ill patients with sepsis-associated acute kidney injury. Journal of Clinical Anesthesia 83, 110960. 10.1016/j.jclinane.2022.110960 36272399

[B9] Iba T. Di Nisio M. Levy J. H. Kitamura N. Thachil J. (2017). New criteria for sepsis-induced coagulopathy (SIC) following the revised sepsis definition: a retrospective analysis of a nationwide survey. BMJ Open 7 (9), e017046. 10.1136/bmjopen-2017-017046 PMC5623518 28963294

[B10] Iba T. Umemura Y. Watanabe E. Wada T. Hayashida K. Kushimoto S. (2019). Diagnosis of sepsis-induced disseminated intravascular coagulation and coagulopathy. Acute Medicine and Surgery 6, 223–232. 10.1002/ams2.411 31304023 PMC6603393

[B11] Inata Y. Piraino G. Hake P. W. O'Connor M. Lahni P. Wolfe V. Schulte C. Moore V. James J. M. Zingarelli B. (2018). Age-dependent cardiac function during experimental sepsis: effect of pharmacological activation of AMP-activated protein kinase by AICAR. American Journal of Physiology. Heart and Circulatory Physiology 315 (4), H826–H837. 10.1152/ajpheart.00052.2018 29979626 PMC6230907

[B12] Johnson A. Bulgarelli L. Pollard T. Horng S. Celi L. A. Mark R. (2023). MIMIC-IV (version 2.2). PhysioNet. 10.13026/6mm1-ek67

[B13] Kai K. Yang G. Wang S. C. Liu H. T. Kong W. L. Zhang X. Huang R. Qi Z. D. Zheng J. B. Qu J. D. Liu R. J. Liu Y. S. Wang H. L. Yu K. J. (2018). Dexmedetomidine protects against lipopolysaccharide-induced sepsis-associated acute kidney injury via an α7 nAChR-dependent pathway. Biomed Pharmacother 106, 210–216. 10.1016/j.biopha.2018.06.059 29960167

[B14] Kang K. Gao Y. Wang S. C. Liu H. T. Kong W. L. Zhang X. Huang R. Qi Z. D. Zheng J. B. Qu J. D. Liu R. J. Liu Y. S. Wang H. L. Yu K. J. (2018). Dexmedetomidine protects against lipopolysaccharide-induced sepsis-associated acute kidney injury via an α7 nAChR-dependent pathway. Biomedicine and Pharmacotherapy 106, 210–216. 10.1016/j.biopha.2018.06.059 29960167

[B45] Kawazoe Y. Miyamoto K. Morimoto T. Yamamoto T. Fuke A. Hashimoto A. (2017). Effect of dexmedetomidine on mortality and ventilator-free days in patients requiring mechanical ventilation with sepsis: a randomized clinical trial. JAMA 317 (13), 1321–1328. 10.1001/jama.2017.2088 28322414 PMC5469298

[B15] La Mura V. Gagliano N. Arnaboldi F. Sartori P. Procacci P. Denti L. Liguori E. Bitto N. Ristagno G. Latini R. Dondossola D. Salerno F. Tripodi A. Colombo M. Peyvandi F. (2022). Simvastatin prevents liver microthrombosis and sepsis-induced coagulopathy in a rat model of endotoxemia. Cells 11 (7), 1148. 10.3390/cells11071148 35406712 PMC8997834

[B16] Levi M. van der Poll T. (2017). Coagulation and sepsis. Thrombosis Research 149, 38–44. 10.1016/j.thromres.2016.11.007 27886531

[B17] Li S. Yang Y. Yu C. Yao Y. Wu Y. Qian L. Cheung C. W. (2015). Dexmedetomidine analgesia effects in patients undergoing dental implant surgery and its impact on postoperative inflammatory and oxidative stress. Oxidative Medicine and Cellular Longevity 2015, 186736. 10.1155/2015/186736 26171113 PMC4485522

[B18] Li X. Li L. Shi Y. Yu S. Ma X. (2020). Different signaling pathways involved in the anti-inflammatory effects of unfractionated heparin on lipopolysaccharide-stimulated human endothelial cells. Journal of Inflammation (London, England) 17, 5. 10.1186/s12950-020-0238-7 32063752 PMC7011532

[B19] Lyons P. G. Micek S. T. Hampton N. Kollef M. H. (2018). Sepsis-associated coagulopathy severity predicts hospital mortality. Critical Care Medicine 46, 736–742. 10.1097/CCM.0000000000002997 29373360

[B20] Ma X. F. Lv S. J. Wei S. Q. Mao B. R. Zhao X. X. Jiang X. Q. Zeng F. Du X. K. (2023). Influences of dexmedetomidine on stress responses and postoperative cognitive and coagulation functions in patients undergoing radical gastrectomy under general anesthesia. World Journal of Gastrointestinal Surgery 15 (6), 1169–1177. 10.4240/wjgs.v15.i6.1169 37405107 PMC10315113

[B21] Mao J. Y. Zhang J. H. Cheng W. Chen J. W. Cui N. (2021). Effects of neutrophil extracellular traps in patients with septic coagulopathy and their interaction with autophagy. Frontiers in Immunology 12, 757041. 10.3389/fimmu.2021.757041 34707618 PMC8542927

[B22] Mei B. Li J. Zuo Z. (2021). Dexmedetomidine attenuates sepsis-associated inflammation and encephalopathy via central α2A adrenoceptor. Brain, Behavior, and Immunity 91, 296–314. 10.1016/j.bbi.2020.10.008 33039659 PMC7749843

[B23] Meng Q. Guo P. Jiang Z. Bo L. Bian J. (2020). Dexmedetomidine inhibits LPS-induced proinflammatory responses via suppressing HIF1α-dependent glycolysis in macrophages. Aging 12 (10), 9534–9548. 10.18632/aging.103226 32433037 PMC7288940

[B24] Morelli A. Sanfilippo F. Arnemann P. Hessler M. Kampmeier T. G. D'Egidio A. (2019). The effect of propofol and dexmedetomidine sedation on norepinephrine requirements in septic shock patients: A crossover trial. Critical Care Medicine 47 (2), e89–e95. 10.1097/CCM.0000000000003520 30394918

[B25] Nakashima T. Miyamoto K. Shima N. Kato S. Kawazoe Y. Ohta Y. Morimoto T. Yamamura H. (2020). Dexmedetomidine improved renal function in patients with severe sepsis: an exploratory analysis of a randomized controlled trial. Journal of Intensive Care 8, 1. 10.1186/s40560-019-0415-z 31908779 PMC6939335

[B26] Ning Q. Liu Z. Wang X. Zhang R. Zhang J. Yang M. Sun H. Han F. Zhao W. Zhang X. (2017). Neurodegenerative changes and neuroapoptosis induced by systemic lipopolysaccharide administration are reversed by dexmedetomidine treatment in mice. Neurological Research 39, 357–366. 10.1080/01616412.2017.1281197 28173746

[B27] Ohta Y. Miyamoto K. Kawazoe Y. Yamamura H. Morimoto T. (2020). Effect of dexmedetomidine on inflammation in patients with sepsis requiring mechanical ventilation: a sub-analysis of a multicenter randomized clinical trial. Critical Care 24 (1), 493. 10.1186/s13054-020-03207-8 32778146 PMC7416813

[B28] Peng J. C. Nie F. Li Y. J. Xu Q. Y. Xing S. P. Li W. (2021). Favorable outcomes of anticoagulation with unfractioned heparin in sepsis-induced coagulopathy: A retrospective analysis of MIMIC-III database. Frontiers in Medicine 8, 773339. 10.3389/fmed.2021.773339 35047524 PMC8761617

[B29] Rudd K. E. Johnson S. C. Agesa K. M. Shackelford K. A. Tsoi D. Kievlan D. R. Colombara D. V. Ikuta K. S. Kissoon N. Finfer S. (2020). Global, regional, and national sepsis incidence and mortality, 1990–2017: analysis for the Global Burden of Disease Study. Lancet 395 (10219), 200–211. 10.1016/S0140-6736(19)32989-7 31954465 PMC6970225

[B30] Sha J. Zhang H. Zhao Y. Feng X. Hu X. Wang C. (2019). Dexmedetomidine attenuates lipopolysaccharide-induced liver oxidative stress and cell apoptosis in rats by increasing GSK-3β/MKP-1/Nrf2 pathway activity via the α2 adrenergic receptor. Toxicology and Applied Pharmacology 364, 144–152. 10.1016/j.taap.2018.12.017 30597158

[B31] Shan X. S. Hu L. K. Wang Y. Liu H. Y. Chen J. Meng X. W. Pu J. X. Huang Y. H. Hou J. Q. Feng X. M. Liu H. Meng L. Peng K. Ji F. H. (2022). Effect of Perioperative Dexmedetomidine on Delayed Graft Function Following a Donation-After-Cardiac-Death Kidney Transplant: A Randomized Clinical Trial. JAMA Network Open 5 (6), e2215217. 10.1001/jamanetworkopen.2022.15217 35657627 PMC9166619

[B32] She H. Zhu Y. Deng H. Kuang L. Fang H. Zhang Z. Duan C. Ye J. Zhang J. Liu L. Hu Y. Li T. (2021). Protective effects of dexmedetomidine on the vascular endothelial barrier function by inhibiting mitochondrial fission via ER/mitochondria contact. Frontiers in Cell and Developmental Biology 9, 636327. 10.3389/fcell.2021.636327 33777946 PMC7991806

[B33] Shin H. J. Boo G. Na H. S. (2021). Effects of dexmedetomidine on blood coagulation: an *in vitro* study using rotational thromboelastometry. Journal of Anesthesia 35 (5), 633–637. 10.1007/s00540-021-02969-x 34268623

[B34] Singer M. Deutschman C. S. Seymour C. W. Shankar-Hari M. Annane D. Bauer M. (2016). The third international consensus definitions for sepsis and septic shock (Sepsis-3). JAMA 315, 801–810. 10.1001/jama.2016.0287 26903338 PMC4968574

[B35] Slim M. A. Turgman O. van Vught L. A. van der Poll T. Wiersinga W. J. (2024). Non-conventional immunomodulation in the management of sepsis. European Journal of Internal Medicine 121, 9–16. 10.1016/j.ejim.2023.10.032 37919123

[B36] Song J. H. Shim H. Y. Lee T. J. Jung J. K. Cha Y. D. Lee D. I. Kim G. W. Han J. U. (2014). Comparison of dexmedetomidine and epinephrine as an adjuvant to 1% mepivacaine in brachial plexus block. Korean Journal of Anesthesiology 66 (4), 283–289. 10.4097/kjae.2014.66.4.283 24851163 PMC4028555

[B37] Sun Y. B. Zhao H. Mu D. L. Zhang W. Cui J. Wu L. (2019). Dexmedetomidine inhibits astrocyte pyroptosis and subsequently protects the brain in *in vitro* and *in vivo* models of sepsis. Cell Death and Disease 10, 167. 10.1038/s41419-019-1416-5 30778043 PMC6379430

[B38] Tao W. H. Shan X. S. Zhang J. X. Liu H. Y. Wang B. Y. Wei X. Zhang M. Peng K. Ding J. Xu S. X. Li L. G. Hu J. K. Meng X. W. Ji F. H. (2022). Dexmedetomidine attenuates ferroptosis-mediated renal ischemia/reperfusion injury and inflammation by inhibiting ACSL4 via α2-AR. Frontiers in Pharmacology 13, 782466. 10.3389/fphar.2022.782466 35873574 PMC9307125

[B39] Xu P. Chen L. Zhu Y. Yu S. Chen R. Huang W. Wu F. Zhang Z. (2022). Critical Care Database Comprising Patients With Infection. Front Public Health 10, 852410. 10.3389/fpubh.2022.852410 35372245 PMC8968758

[B40] Xu P. Chen L. Zhang Z. (2022). Critical care database comprising patients with infection at Zigong Fourth People's Hospital (version 1.1). PhysioNet. 10.13026/xpt9-z726 PMC8968758 35372245

[B41] Yang J. Zhang B. Hu C. Jiang X. Shui P. Huang J. Hong Y. Ni H. Zhang Z. (2024). Identification of clinical subphenotypes of sepsis after laparoscopic surgery. Laparosc Endosc Robot Surg 7, 16–26. 10.1016/j.lers.2024.02.001

[B42] Yu Q. Li Q. Yang X. Liu Q. Deng J. Zhao Y. Hu R. Dai M. (2021). Dexmedetomidine suppresses the development of abdominal aortic aneurysm by downregulating the mircoRNA‑21/PDCD 4 axis. International Journal of Molecular Medicine 47 (5), 90. 10.3892/ijmm.2021.4923 33786608 PMC8029612

[B43] Zhang Z. Zhang G. Goyal H. Mo L. Hong Y. (2018). Identification of subclasses of sepsis that showed different clinical outcomes and responses to amount of fluid resuscitation: a latent profile analysis. Crit Care 22 (1), 347. 10.1186/s13054-018-2279-3 30563548 PMC6299613

[B44] Zhao S. Zhou R. Zhong Q. Zhang M. (2024). Effect of age and ICU types on mortality in invasive mechanically ventilated patients with sepsis receiving dexmedetomidine: a retrospective cohort study with propensity score matching. Frontiers in Pharmacology 15, 1344327. 10.3389/fphar.2024.1344327 38487173 PMC10937464

