# Supplementary figures and images for: The relationship between dexmedetomidine administration and prognosis in patients with sepsis-induced coagulopathy: a retrospective cohort study
Source: Front Pharmacol. 2024 Jul 23;15:1414809. doi: 10.3389/fphar.2024.1414809 (PMC11300284; doi:10.3389/fphar.2024.1414809)

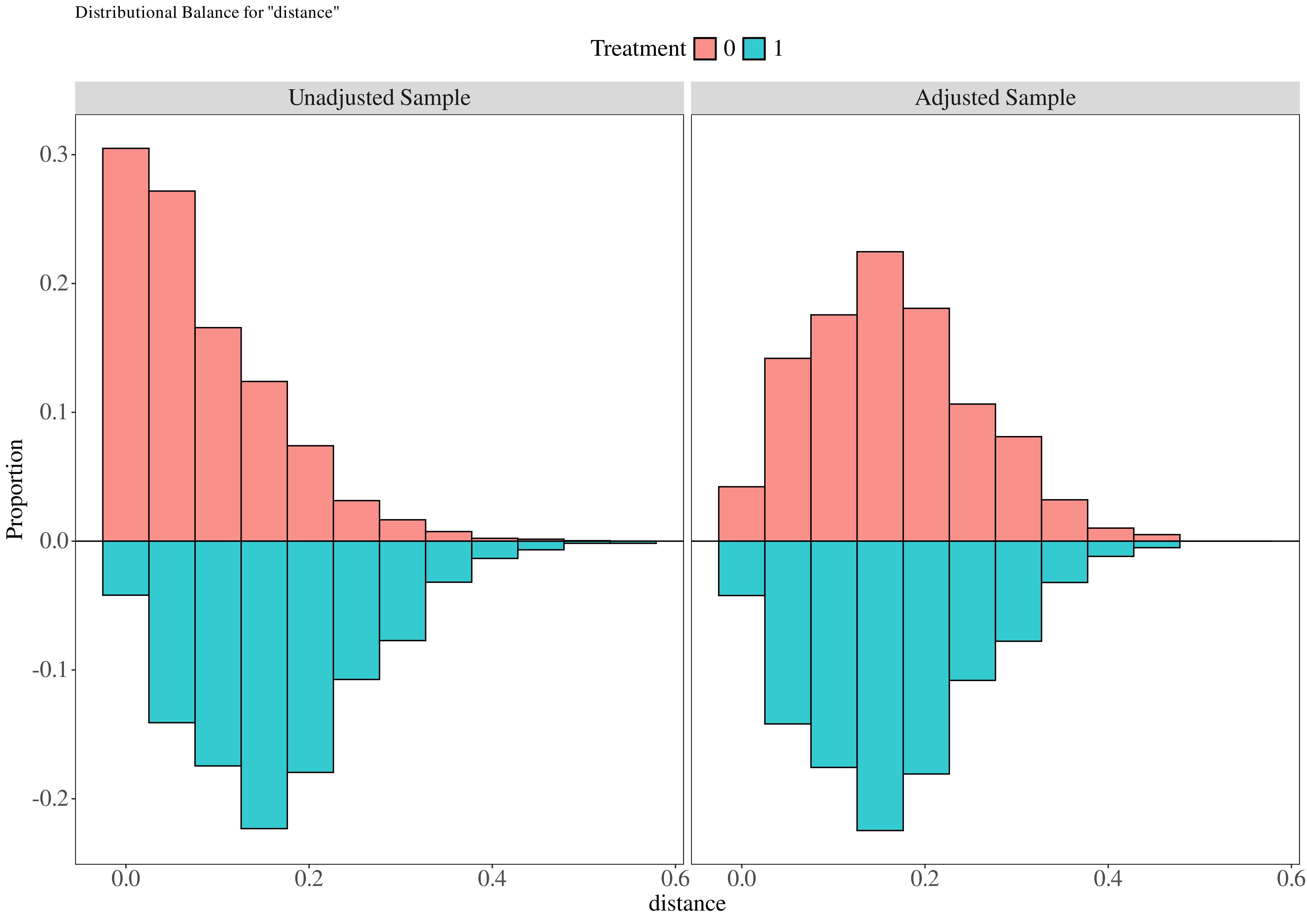

Supplement: Supplementary file 1 [file Image3.JPEG]

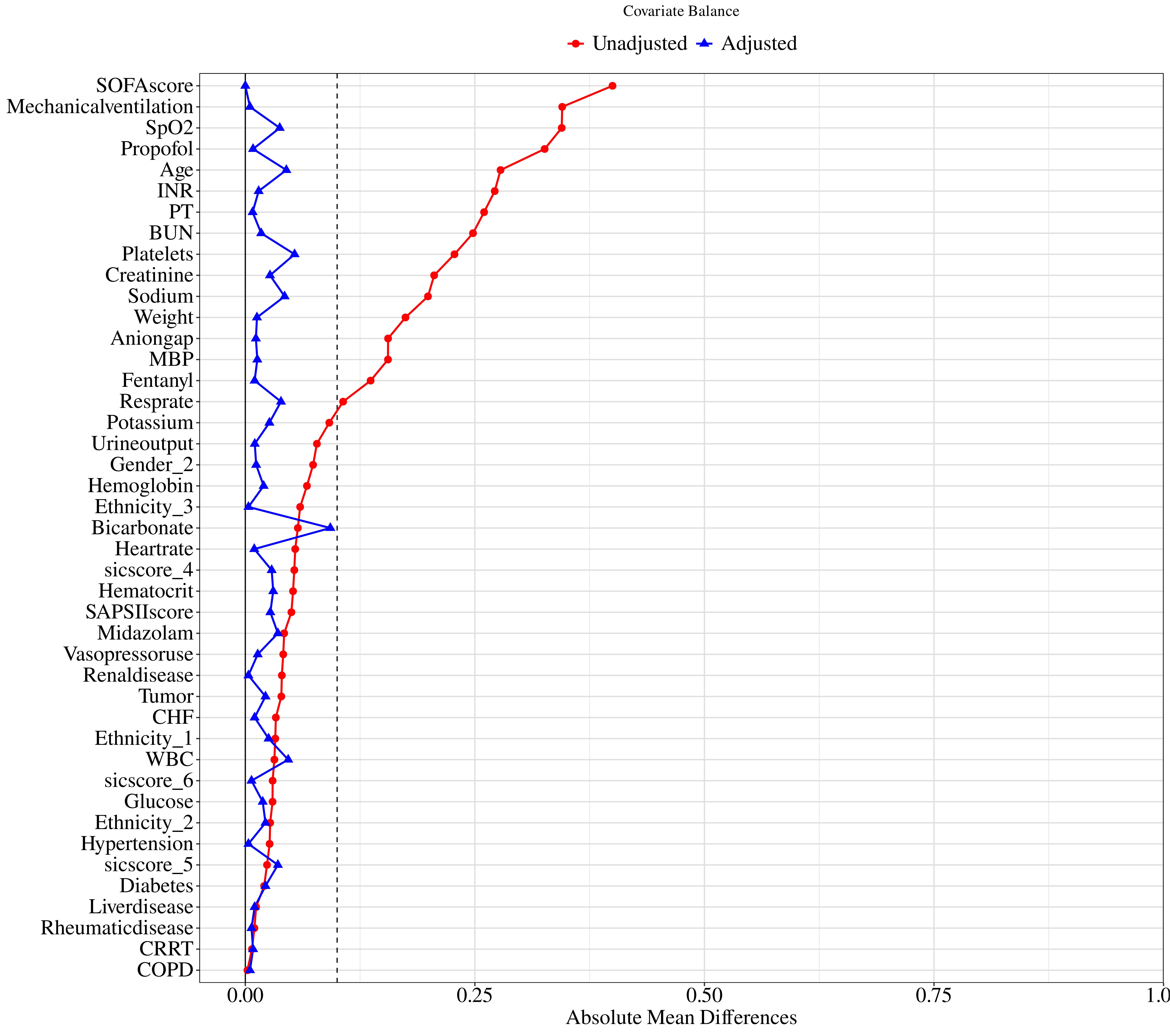

Supplement: Supplementary file 3 [file Image1.JPEG]

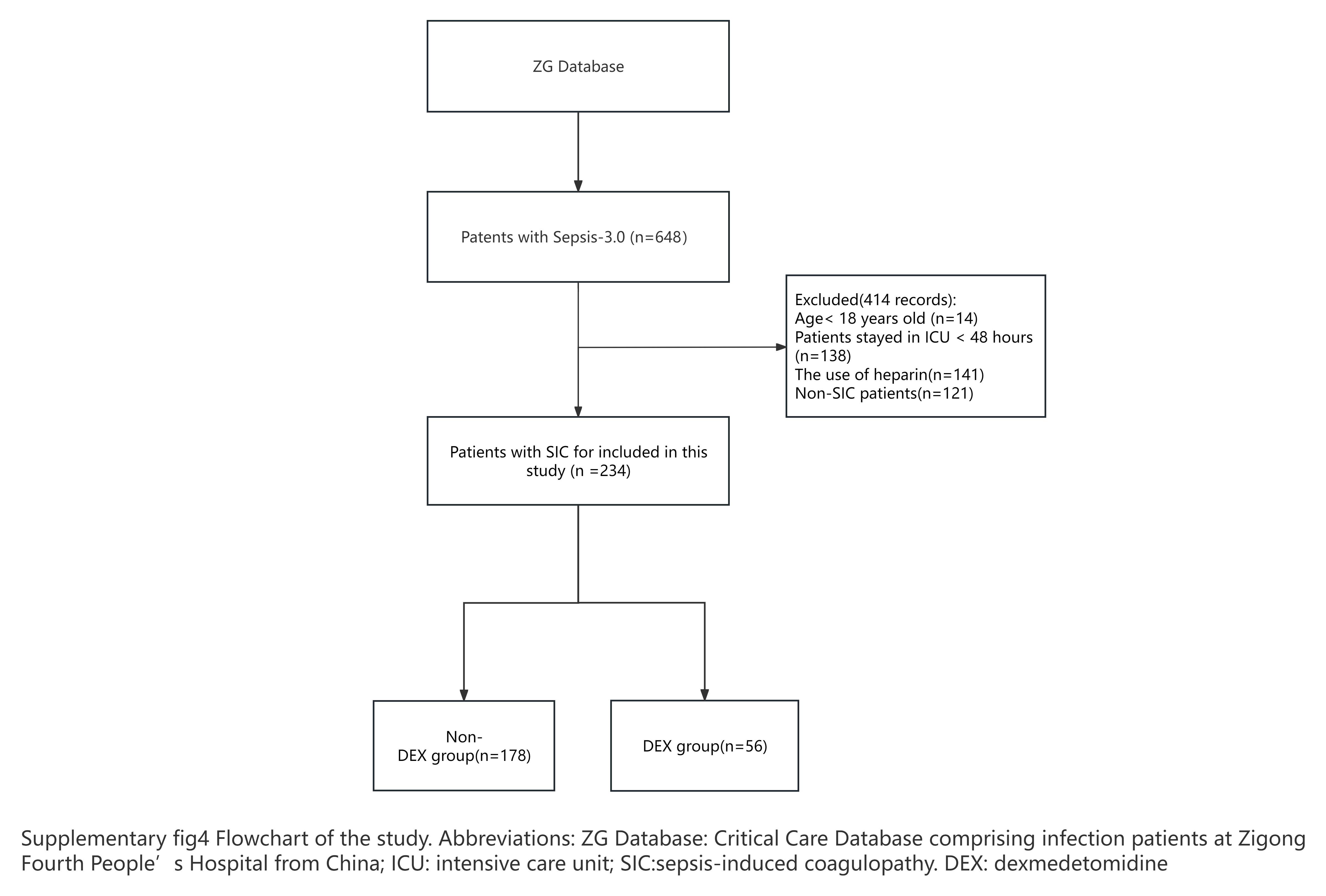

Supplement: Supplementary file 4 [file Image4.JPEG]

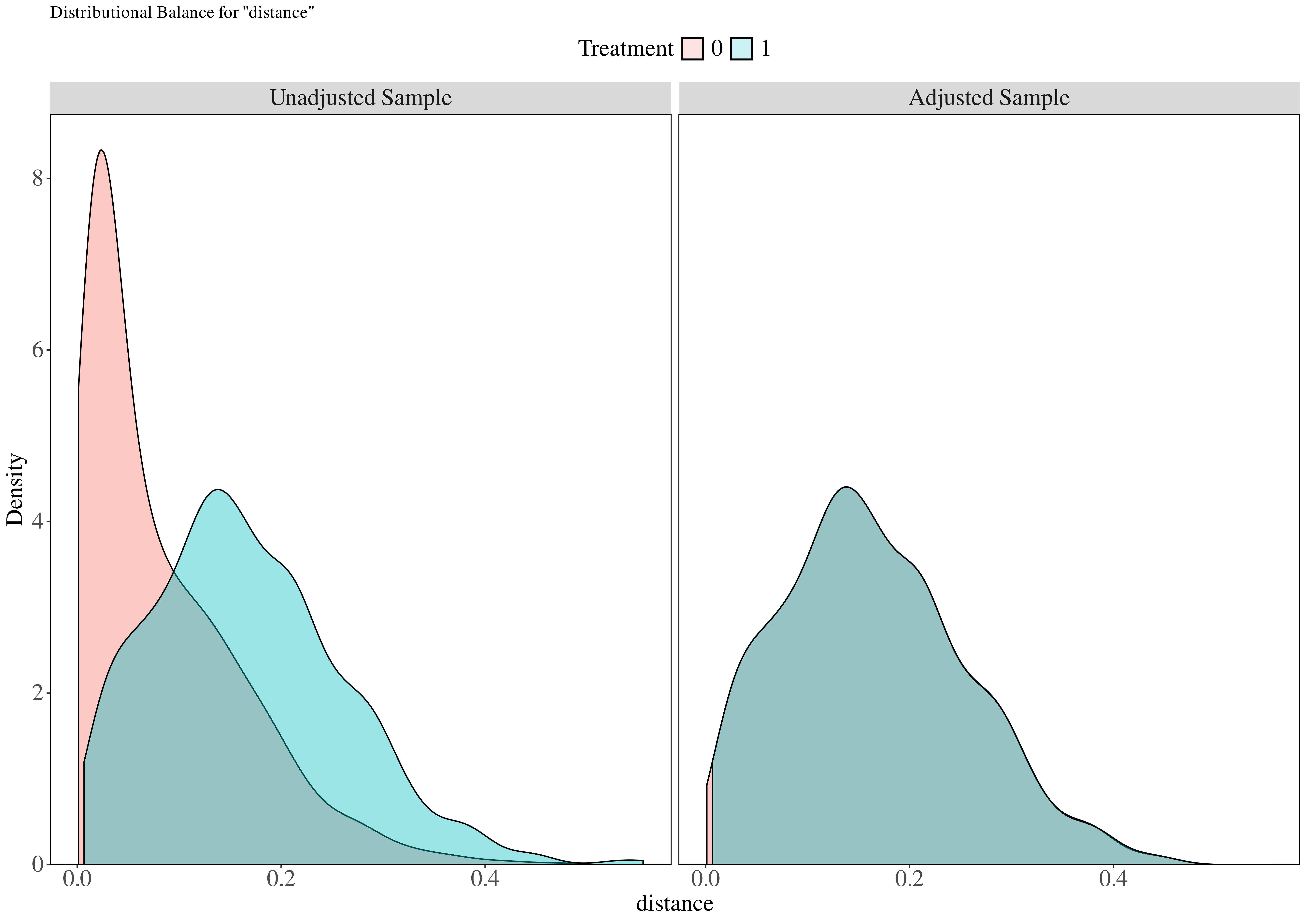

Supplement: Supplementary file 5 [file Image2.JPEG]

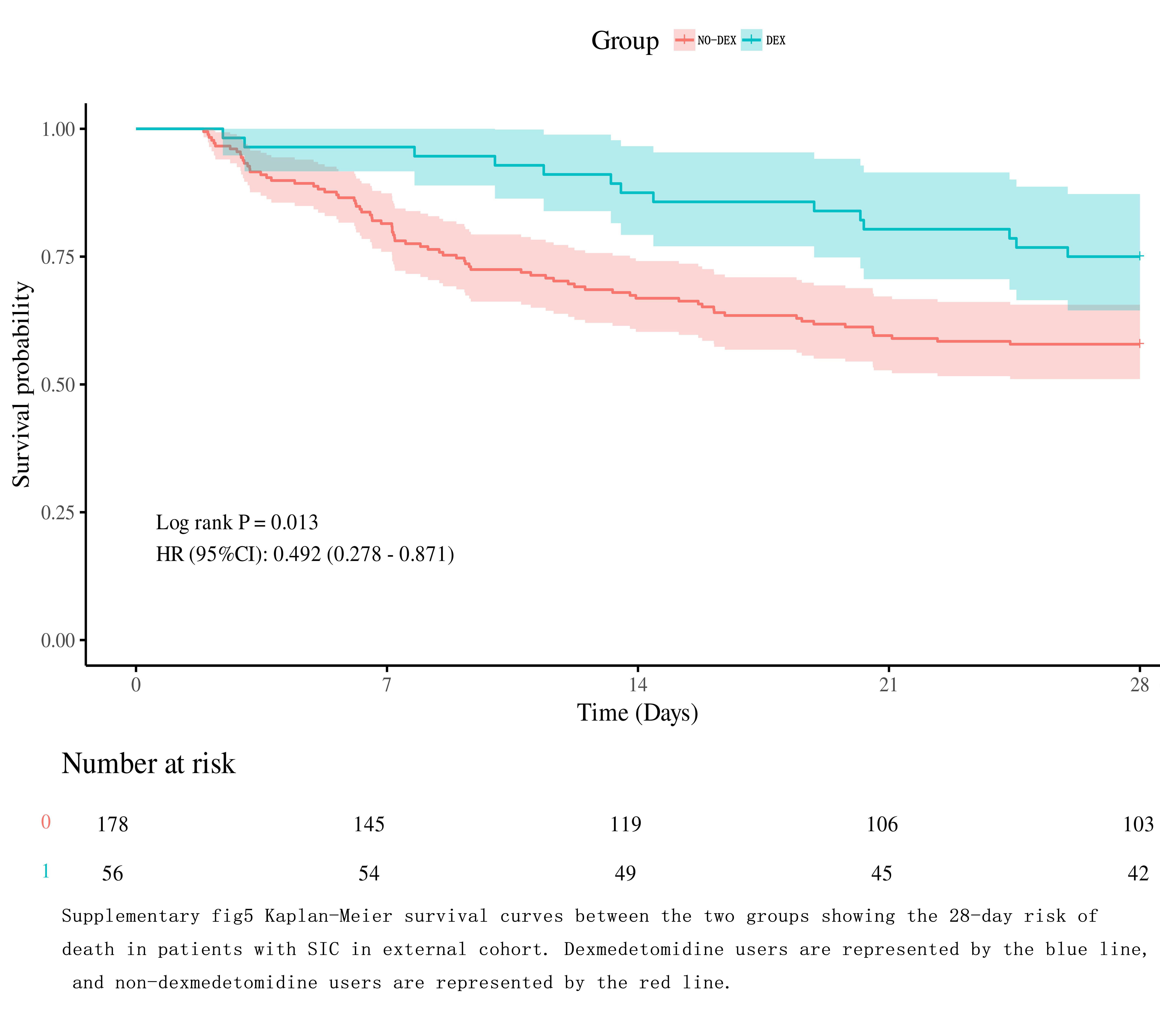

Supplement: Supplementary file 6 [file Image5.JPEG]
