# Supplementary material for: The relationship between dexmedetomidine administration and prognosis in patients with sepsis-induced coagulopathy: a retrospective cohort study
Source: Front Pharmacol. 2024 Jul 23;15:1414809. doi: 10.3389/fphar.2024.1414809 (PMC11300284; doi:10.3389/fphar.2024.1414809)
Supplement: Supplementary file 2 [file Table1.DOCX]

***Supplementary1 materials***

**Table S1 diagnosis criteria of sepsis-induced coagulopathy**

|  | **points** | **items** |
| --- | --- | --- |
| Platelet count (10^9^/L) | 2 | < 100 |
|  | 1 | ≥ 100, <150 |
| INR | 2 | > 1.4 |
|  | 1 | >1.2, ≤1.4 |
| Total SOFA score | 2 | ≥ 2 |
|  | 1 | 1 |

Abbreviations: INR, international normalized ratio.

Note: total SIC score is 4 or more with sum of SOFA score and coagulation criteria exceeding 2. Total SOFA score is the sum of four items(respiratory SOFA, cardiovascular SOFA, hepatic SOFA, and renal SOFA).

Table S2. Missing number (%) for included variables in the datasets

| **Variable** | **MissingCount** | **MissingPercent** |
| --- | --- | --- |
| Age | 0 | 0% |
| Gender | 0 | 0% |
| Urine output | 223 | 3.43% |
| Weight | 42 | 0.65% |
| SOFA score | 0 | 0% |
| SAPSII score | 0 | 0% |
| Ethnicity | 0 | 0% |
| Mechanical ventilation | 0 | 0% |
| Vasopressor use | 0 | 0% |
| CRRT | 0 | 0% |
| Hypertension | 0 | 0% |
| CHF | 0 | 0% |
| COPD | 0 | 0% |
| Rheumatic disease | 0 | 0% |
| Liver disease | 0 | 0% |
| Diabetes | 0 | 0% |
| Renal disease | 0 | 0% |
| Tumor | 0 | 0% |
| Heart rate | 7 | 0.11% |
| MBP | 8 | 0.12% |
| Resp rate | 10 | 0.15% |
| SpO2 | 9 | 0.14% |
| Hematocrit | 0 | 0% |
| Hemoglobin | 1 | 0.02% |
| Platelets | 0 | 0% |
| WBC | 2 | 0.03% |
| Anion gap | 17 | 0.26% |
| Bicarbonate | 2 | 0.03% |
| BUN | 1 | 0.02% |
| Creatinine | 1 | 0.02% |
| Sodium | 4 | 0.06% |
| Potassium | 12 | 0.18% |
| INR | 0 | 0% |
| PT | 0 | 0% |
| Glucose | 7 | 0.11% |
| sicscore | 0 | 0% |
| Propofol | 0 | 0% |
| Midazolam | 0 | 0% |
| Fentanyl | 0 | 0% |

Abbreviations: SOFA score,Sequential Organ Failure Assessment Score; SAPSII score - Simplified Acute Physiology Score II; CRRT - Continuous Renal Replacement Therapy; MBP,Mean Blood Pressure; Resp Rate,Respiratory Rate; SpO2,Saturation of Peripheral Oxygen; WBC,White Blood Cell count; BUN,Blood Urea Nitrogen; INR,International Normalized Ratio; PT,Prothrombin Time

Table S3. Cox regression model after propensity score matching in patients with SIC

| **Variables** | **HR (univariable)** | **HR (multivariable)** |
| --- | --- | --- |
| **Age** | 1.01 (1.00-1.02, p=0.078) |  |
| **Gender** |  |  |
| Male | reference |  |
| Female | 1.22 (0.92-1.63, p=0.171) |  |
| **Ethnicity** |  |  |
| White | reference |  |
| Black | 1.15 (0.66-2.01, p=0.619) | 1.07 (0.61-1.88, p=0.816) |
| Other | 1.87 (1.39-2.51, p<.001) | 1.69 (1.25-2.29, p=0.001) |
| **Heart rate** | 1.01 (1.00-1.02, p=0.017) | 1.00 (0.99-1.01, p=0.895) |
| **Resp rate** | 1.08 (1.04-1.11, p<.001) | 1.03 (0.99-1.07, p=0.150) |
| **SpO2** | 0.86 (0.80-0.93, p<.001) | 0.92 (0.85-1.00, p=0.054) |
| **Anion gap** | 1.10 (1.08-1.13, p<.001) | 1.04 (1.00-1.08, p=0.033) |
| **Bicarbonate** | 0.94 (0.92-0.97, p<.001) | 1.01 (0.98-1.04, p=0.510) |
| **BUN** | 1.02 (1.01-1.02, p<.001) | 1.01 (1.00-1.02, p=0.001) |
| **Creatinine** | 1.21 (1.11-1.32, p<.001) | 1.01 (0.88-1.17, p=0.848) |
| **CRRT** |  |  |
| No | reference |  |
| Yes | 2.30 (1.55-3.41, p<.001) | 1.08 (0.68-1.70, p=0.750) |
| **Vasopressor use** |  |  |
| No | reference |  |
| Yes | 2.08 (1.51-2.87, p<.001) | 1.51 (1.04-2.17, p=0.028) |
| **Propofol** |  |  |
| No | reference |  |
| Yes | 0.64 (0.45-0.90, p=0.010) | 0.89 (0.62-1.27, p=0.510) |
| **Midazolam** |  |  |
| No | reference |  |
| Yes | 1.92 (1.45-2.53, p<.001) | 1.46 (1.09-1.96, p=0.011) |
| **Fentanyl** |  |  |
| No | reference |  |
| Yes | 0.92 (0.69-1.21, p=0.545) |  |
| **Liver disease** |  |  |
| No | reference |  |
| Yes | 1.57 (1.33-1.85, p<.001) | 1.44 (1.17-1.76, p=0.001) |
| **Tumor** |  |  |
| No | reference |  |
| Yes | 1.78 (1.46-2.18, p<.001) | 1.58 (1.27-1.96, p<.001) |
| **SIC score** |  |  |
| 4 | reference |  |
| 5 | 0.82 (0.57-1.20, p=0.310) | 0.77 (0.52-1.12, p=0.171) |
| 6 | 1.47 (1.07-2.01, p=0.016) | 0.98 (0.69-1.41, p=0.933) |
| **Dexmedetomidine** |  |  |
| No | reference |  |
| Yes | 0.70 (0.53-0.93, p=0.013) | 0.71 (0.53-0.95, p=0.020) |

**Abbreviations: Abbreviations: PSM: propensity score matching; DEX: dexmedetomidine; SMD: standardized mean difference,SOFA: Sequential Organ Failure Assessment,SAPS II: Simplified Acute Physiology Score II,ICU: Intensive Care Unit,MAP: Mean Arterial Pressure,WBC: White Blood Cell Count,INR: International Normalized Ratio,PT: Prothrombin Time,APTT: Activated Partial Thromboplastin Time,SIC: Sepsis-Induced Coagulopathy,CRRT - Continuous Renal Replacement Therapy;Resp Rate,Respiratory Rate; SpO2,Saturation of Peripheral Oxygen;BUN,Blood Urea Nitrogen; HR, hazard ratio; OR,Odds Ratio;CI, confidence interval.**
