# Supplementary material for: The relationship between dexmedetomidine administration and prognosis in patients with sepsis-induced coagulopathy: a retrospective cohort study
Source: Front Pharmacol. 2024 Jul 23;15:1414809. doi: 10.3389/fphar.2024.1414809 (PMC11300284; doi:10.3389/fphar.2024.1414809)
Supplement: Supplementary file 7 [file Table2.DOCX]

***Supplementary materials2***

| **Table S1: Baseline Characteristics in external cohort** | | | |
| --- | --- | --- | --- |
| **Characteristics** | **Non-DEX group(n = 178)** | **DEX group(n = 56)** | **p** |
| Age (years) | 70.00 (63.00, 81.75) | 69.00 (61.00, 77.00) | 0.146 |
| Gender [female, n (%)] | 63 (35.39) | 17 (30.36) | 0.488 |
| Propofol , n (%) | 36 (20.22) | 3 (5.36) | 0.009 |
| Sufentanil, n (%) | 104 (58.43) | 43 (76.79) | 0.013 |
| SIC score, n (%) |  |  | 0.144 |
| 4 | 66 (37.08) | 28 (50.00) |  |
| 5 | 48 (26.97) | 15 (26.79) |  |
| 6 | 64 (35.96) | 13 (23.21) |  |
| 28-day mortality, n(%) | 75 (42.13) | 14 (25.00) | 0.021 |
| The length of hospital stay (d) | 13.59 (5.70, 24.31) | 23.96 (13.78, 42.74) | <0.001 |
| The length of ICU stay (d) | 6.79 (3.59, 14.63) | 15.47 (7.09, 25.16) | <0.001 |

Abbreviations: SIC: Sepsis-Induced Coagulopathy;DEX: dexmedetomidine; ICU: Intensive Care Unit

**Table S2:Survival results of dexmedetomidine and non-user groups in SIC in external cohort**

| **Categories** | **28-day mortality** | |
| --- | --- | --- |
| Before PSM | HR (95% CI) | P-value |
| Model1 | 0.49 (0.28-0.87) | 0.015 |
| Model2 | 0.47 (0.27-0.84) | 0.01 |
| Model3 | 0.49 (0.27-0.89) | 0.019 |
| Model 1: unadjusted | | |
| Model 2: adjusted for age, gender | | |
| Model 3: adjusted for Cox regression were used to estimate the impact of dexmedetomidine use on mortality outcomes, adjusting for confounding variables selected based on P value <0.05 in univariate analysis, including age, gender,propofol,sufentanil | | |

Abbreviations: SIC: Sepsis-Induced Coagulopathy;DEX: dexmedetomidine; ICU: Intensive Care Unit
